# The Ohm Law as an Alternative for the Entropy Origin Nonlinearities in Conductivity of Dilute Colloidal Polyelectrolytes

**DOI:** 10.3390/e22020225

**Published:** 2020-02-17

**Authors:** Ioulia Chikina, Valeri Shikin, Andrey Varlamov

**Affiliations:** 1 LIONS, NIMBE, CEA, CNRS, Universitè Paris-Saclay, CEA Saclay, 91191 Gif-sur-Yvette, France; julia.chikina@cea.fr; 2 ISSP, RAS, Chernogolovka, 142432 Moscow, Russia; shikin@issp.ac.ru; 3 CNR-SPIN, c/o DICII-Universitá di Roma Tor Vergata, Via del Politecnico, 1, 00133 Roma, Italy

**Keywords:** polyelectrolytes, Ohm law, colloids

## Abstract

We discuss the peculiarities of the Ohm law in dilute polyelectrolytes containing a relatively low concentration $n_{⊙}$ of multiply charged colloidal particles. It is demonstrated that in these conditions, the effective conductivity of polyelectrolyte is the linear function of $n_{⊙}$. This happens due to the change of the electric field in the polyelectrolyte under the effect of colloidal particle polarization. The proposed theory explains the recent experimental findings and presents the alternative to mean spherical approximation which predicts the nonlinear I–V characteristics of dilute colloidal polyelectrolytes due to entropy changes.

## 1. Introduction

Polyelectrolytes are polymers whose repeating units contain a group of electrolytes. These groups dissociate in aqueous solutions, making the polymers charged. Polyelectrolyte properties resemble those of both electrolytes and polymers, and, like salts, their solutions are electrically conductive. The incorporation of the nano- and micro-meter-sized charged colloidal particles can dramatically change the electrical and heat transport properties of such systems. For instance, the authors of Ref. [1] study the electrical transport in charged colloidal suspensions of iron oxide nanoparticles (maghemite) dispersed in an aqueous medium, while in Ref. [2], the thermal and electrical transport is investigated in ionically stabilized magnetic nanoparticles dispersed in aqueous potassium ferro/ferricyanide electrolytes. Both groups report the unusual effect of multiply charged colloidal particles on conductivity of the dilute polyelectrolytes. It turns out that the latter grows linearly with an increase of colloidal particle concentration.

This finding seems to be non-trivial from the point of view of the percolation theory (see, for example, [3]). Indeed, in accordance with the latter, the conductivity of a mixture between dielectric (in our case water molecules) and conducting (colloidal particle with counter-ions coat) components remains minute until the fraction of the conducting phase approaches the percolation threshold, and only in the vicinity of the latter, the conductivity growths smoothly have a value of dielectric component that is similar to to that of a metallic one.

**Figure 1:**
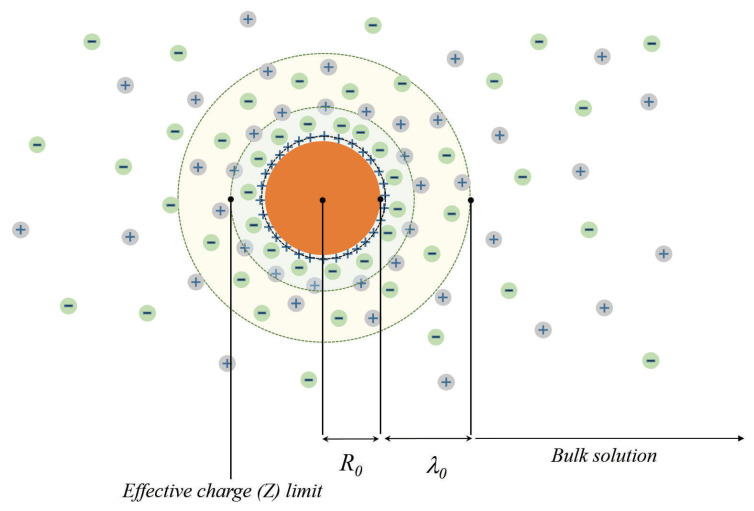
The schematic presentation of the multiply charged colloidal particle surrounded by the cloud of counter-ions.

Before discussing this contradiction, let us make an excursus into the physics of semiconductors. In the theory of semiconductors [3], the regions of weak and strong doping (i.e., introduction of charged impurities or structural defects with the purpose of changing the electrical properties of a semiconductor) are distinguished. In the low doping regime, the impurity concentration $n_{⊙}$ is so small that the distances between them significantly exceed the Debye length $\lambda_{0}$ and the bare radius of the colloidal particle $R_{0}$, i.e.

(1) $$n_{⊙}{\left(\lambda_{0}+R_{0}\right)}^{3}\ll 1,\;R_{0}\leq \lambda_{0},$$

and the intrinsic charge carriers of semiconductor completely screen the electric fields produced by the charged impurities (see Figure 1). In the strong doping regime, when criterion (1) is violated, the fields produced by the dopants are screened only partially and their interaction becomes significant.

Returning to the case of the dilute colloidal polyelectrolyte, one can map its properties to the ones of the weak doped semiconductor and identify $n_{⊙}$ with the concentration of the colloidal particles, while $\lambda_{0}$ should be related to their characteristic size. The latter is determined by the known concentration $n_{0}$ of the counterions of the electrolyte hosting charged colloidal particles.

The criterion (1) is in a reasonable agreement to the common concepts of the physics of dilute polyelectrolytes developed in the 40s of the last century by Derjaguin, Landau, Verwey, and Overbeek [4,5] and known as DLVO formalism. Namely, if the colloidal particles are neutral, they are not stationary in dilute solution and coagulating due to van der Waals forces acts between them. In order to prevent such coagulation processes, one should immerse individual colloidal particles in the electrolyte specific for each sort of them. The latter are called stabilizing electrolytes.

Being immersed (or synthesized within) in an electrolyte solution, the nanoparticles acquire surface ions (e.g., hydroxyl groups, citrate, etc. [6,7,8]) resulting in a very large structural charge eZ (|Z|≫10). Its sign can be both positive or negative, depending on the surface group type. The latter, in return, attracts counterions from the surrounding solvent creating an electrostatic shielding coat of the size $\lambda_{0}$ with an effective charge −eZ. In these conditions, nano-particles approaching between them to the distances $r\leq \lambda_{0}$ begin to repel each other without floculation [4,5,9]. The region of an essential interaction between them in terms of the criterion (1) corresponds to the condition

(2) $$n_{⊙}^{c}{\left(\lambda_{0}+R_{0}\right)}^{3}∼1.$$

In Ref. [1,2], the massive multiply charged colloidal particles are surrounded by the clouds of counter-ions screening their positive charge. Such formations, according to Ref. [3], should not affect the conductivity of the dilute polyelectrolyte until the shells of the neighbor charged complexes do not overlap among themselves (see Equation (2)). The results of both Ref. [1] and [2] demonstrate the opposite: the conductivity of dilute colloidal polyelectrolyte grows linearly with increase of concentration already in the range $n_{⊙}\ll n_{⊙}^{c}$, where there is not yet place for percolation effects.

This contradiction can be removed by noticing that the presence of the multiply charged colloidal particles has an effect not only on the value of conductivity of a solution but also on the local value of the electric field:

(3) $$j\left(n_{⊙}\right)=\sigma \left(n_{⊙}\right)E\left(n_{⊙}\right).$$

It is important to note that the factors in Equation (3) are affected by the presence of the multiply charged colloidal particles in different ways. While the conductivity of the electrolyte at low concentrations of multiply charged colloidal particles ($n_{⊙}\leq n_{⊙}^{c}$) remains almost unchanged, their effect on the local electric field in this range of concentrations is essential. This happens due to polarization of the colloidal particles by an external electric field which, in accordance with the Le Chatelier’s principle, results in the decrease of the effective value of the field. Consequently, the growth of conductivity [1,2] as a function of concentration $n_{⊙}$ is observed in experiments. When the concentration of multiply charged colloidal particles reaches the percolation threshold ($n_{⊙}=n_{⊙}^{c}$), the role of the factors in Equation (3) is reversed. Here, the subsystem of colloidal particles forms clusters and cannot be considered more as the gas of polarized highly conducting particles. Yet, in this range of concentrations, the new channel of percolation charge transfer is opened and the total conductivity of the electrolyte growth further increase by $n_{⊙}$.

The state-of-the-art in transport phenomena in polyelectrolytes was recently reviewed in Ref. [1]. Focusing mainly on the results of the microscopic approach (so called mean spherical approximation theory (MSA)) [10,11,12], the authors discuss mobility, diffusion coefficient, and the effective charge space distribution of the colloidal particles as the function of their concentration. Yet, in Ref. [1], there is not any information concerning the effect of clusters of polarization on the charge transfer process in such complex systems. This aspect of the problem is the subject of our work.

## 2. Effective Electric Field in Bulk of Colloidal Polyelectrolyte

The colloidal polyelectrolyte is a weakly conducting liquid with the small but finite fraction of relatively highly (due to Z≫1) conducting inclusions, i.e., colloidal particles. The collective polarization of these inclusions occurs when the external electric field $E_{0}$ is applied. This phenomenon is analogous to polarization of neutral atoms in gas. The only difference is that the neutral atoms reside in vacuum, while the charged conducting clusters of colloidal polyelectrolyte are immersed in a less, but still conducting, medium. Hence, our goal is to account for this peculiarity and find the effective field which governs the charge transport in such a complex system.

### 2.1. Electric Field in Absence of Current

The space distribution of the effective electric field of the colloidal particle is determined by the Poisson equation (see [3,9])

(4) $$\Delta \varphi =\frac{4\pi }{\epsilon }\rho \left(r\right),\;\rho \left(r\right)=\left|e\right|\left[n_{+}\left(r\right)-n_{-}\left(r\right)\right],$$

where ϵ is the dielectric permittivity of stabilizing electrolyte.

The concentrations of the screening counterions $n_{\pm }\left(r\right)$ is determined self-consistently via the value of local electrostatic potential

(5) $$n_{\pm }\left(r\right)=n_{0}\exp\left[e_{\pm }\varphi \left(r\right)/T\right],$$

$n_{0}=n_{0}^{+}=n_{0}^{-}$ is the counterions bare concentration, occuring due to the complete dissociation of the electrolyte which stabilizes the gas of colloidal particles.

In assumption eφ(r)<T the Poisson equation can be linearized and takes form

(6) $$\Delta \varphi =\varphi /\lambda_{0}^{2},\;\lambda_{0}^{-2}=\frac{8\pi e^{2}}{\epsilon T}n_{0}.$$

This equation should be solved accounting for the boundary conditions

(7) $$r\varphi {\left(r\right)}_{|r\to R_{0}}\to Z\left|e\right|,\;\varphi {\left(r\right)}_{|r\to \infty }\to 0,$$

what results in the standard screened Coulomb potential:

(8) $$\varphi \left(r\right)=Ze\frac{\exp(-\frac{r}{\lambda_{0}})}{r}.$$

The values *Z*, $R_{0}$ and $n_{0}$ of the electrolyte, which stabilizes the colloidal solution can be determined by independent experiments (for example, by measurements of the electrophoretic forces, osmotic pressure, etc. [1]).

One should remember that even strongly diluted polyelectrolytes can undergo the transition to the state of a Wigner crystal in the case of strongly charged colloidal particles (Z≫1). For description of this, observed experimentally [13,14,15], phenomenon the authors of [16] assumed that the interaction between two colloidal particles has the same form of Yukawa potential (8), yet with the renormalized effective charge $Z^{*}\ll Z,$ explicitly depending on the colloidal particles density $n_{⊙}$. The value of $Z^{*}$ is determined in the Wigner-Seitz model from the new boundary condition

$$\frac{\partial \varphi }{\partial r}{|}_{r\to n_{⊙}^{-1/3}}=0$$

replacing that ones, valid for the isolated charged particle in the screening media (see Equation (7)). For some range of the colloidal particles densities $n_{⊙}$ the conditions Z≫1 and $Z^{*}\ll 1$ can be satisfied simultaneously. The former characterizes the properties of the multiply charged colloidal particles, while the latter is determined by the strength of their interaction and $n_{⊙}$. In the range of densities $n_{⊙}$ satisfying Equation (1), the effect of the effective charge $Z^{*}$ on the Ohmic transport is negligible.

### 2.2. Electric Field in Presence of Current

When a stationary current flows through the polyelectrolyte, an internal electric field $\vec{E}$ appears in it. In the approximation of a very diluted solution, one can start considerations from the effect of presence of the isolated colloidal particle on a flowing current. Namely, one should find the perturbation of the internal electric field which would provide the homogeneity of the transport current far from the colloidal particle. A corresponding problem recalls that one of classic hydrodynamics: calculus of the associated mass of the particle moving in the ideal liquid [17].

We choose the center of spherical coordinates coinciding with the colloidal particle and direct the z-axis along the electric field $\vec{E_{0}}$. We assume that the conductivity of the electrolyte in the absence of colloidal particles is $\sigma_{0}$. The highly charged colloidal particle we will model as the conducting solid sphere of the radius $R≃(R_{0}+\lambda_{0})$(see Figure 1) with conductivity $\sigma_{⊙}>\sigma_{0}$. Analysis of the charge transport in multi-phase systems (see [18]) is based on the requirements

(9) $$div\vec{j}=0,\;\vec{j}=\sigma \vec{E}.$$

When the medium conductivity is invariable in space the constancy of the current, this automatically means the homogeneity of the electric field. The situation changes when the system is inhomogeneous and σ≠const. The continuity Equation (9) in this case should be solved with the boundary conditions accounting for the current flow through the boundaries between domains of diverse conductivity. According to Ref. [18,19], the tangential components of electric field intensity at the boundary must be continuous, while the normal ones provide the continuity of the charge transfer. Applying these rules to our simple model of the highly charged colloidal particle in the less conductive medium, one can write

(10) $$j_{n}^{0}=J_{n}^{⊙},\;or\;\sigma_{0}E_{0}=\sigma_{⊙}E_{⊙}.$$

Solution of the system of Equations (9) and (10) for the electrostatic potential in the vicinity of the colloidal particle (r≥R) acquires the form:

(11) $$\varphi \left(r,\theta \right)=-E_{0}r\cos\theta +\left(\frac{\gamma -1}{\gamma +2}\right)E_{0}\frac{R^{3}}{r^{2}}\cos\theta ,$$

with $\gamma =\sigma_{⊙}/\sigma_{0}$. In the limit γ→1 the electric field remains unperturbed, $\vec{E}=-\nabla \varphi \to {\vec{E}}_{0}$. In the opposite case, γ≫1, the dipole perturbation takes the form corresponding to the case of metallic inclusion of the radius *R* in the weakly conducting environment (Ref. [18]):

(12) $$\varphi \left(r,\theta \right)=-E_{0}r\cos\theta \left(1-\frac{R^{3}}{r^{3}}\right).$$

One can see that in accordance with the intuitive expectations, the presence of an isolated colloidal particle in an electrolyte leads to the appearance of the local perturbation of the electric field of the dipole type $\nabla \varphi \propto r^{-3}$ with the value of the dipole moment of one colloidal particle

(13) $$p_{⊙}=\left(\frac{\gamma -1}{\gamma +2}\right)R^{3}E_{0}.$$

Returning to the initial problem of the rarefied gas of colloidal particles of concentration $n_{⊙}$ in the electrolyte media, one can introduce the effective dielectric permittivity $\epsilon_{⊙}$. It can be related to the dipole moment (13) by means of the Clausius–Mossotti relation (see Ref. [18]) and in terms of the material parameters of the problem which is read as:

(14) $$\epsilon_{⊙}=1+4\pi \left(\frac{\gamma -1}{\gamma +2}\right)R^{3}n_{⊙}.$$

One can try to make the model of colloidal particles more realistic assuming that the latter has the structure of a thick-walled sphere; a “nut” with the conducting shell and the insulating core of the bare radius $R_{0}$. This intricacy leads to the change in the expression for the corresponding dipole momentum: instead of Equation (13) it takes the form (see Ref. [18])

(15) $${\tilde{p}}_{⊙}=\frac{\left(2\gamma +1\right)\left(\gamma -1\right)}{\left(2\gamma +1\right)\left(\gamma +2\right)-2{\left(\gamma -1\right)}^{2}R_{0}^{3}/R^{3}}\left(R^{3}-R_{0}^{3}\right)E_{0}.$$

This formula contains two geometrical parameters: *R* and $R_{0}$. The latter should be determined from some independent measurements. The difference $R-R_{0}$ can be identified with the Debye length $\lambda_{0}$ or to consider it as the fitting parameter.

## 3. Ohmic Transport in a Weak Colloidal Polyelectrolyte

Equation (14) demonstrates that growth of the nano-particle concentration $n_{⊙}$ leads to increase of the dielectric constant $\epsilon_{⊙}$, which, in its turn, results in the decrease of the effective electric field in an electrolyte. The latter, in conditions of the fixed transport current, is perceived as the growth of conductivity with an increase of the colloidal particles concentration:

(16) $$\sigma \left(n_{⊙}\right)\;=\;j\epsilon_{⊙}/E_{0}\;=\;\sigma_{0}\;\left[1\;+\;4\pi n_{⊙}\frac{p_{⊙}\left(E_{0}\right)}{E_{0}}\right].$$

This expression can be already used for the experimental data processing.

### 3.1. Approximation of the Conducting Spheres

**Figure 2:**
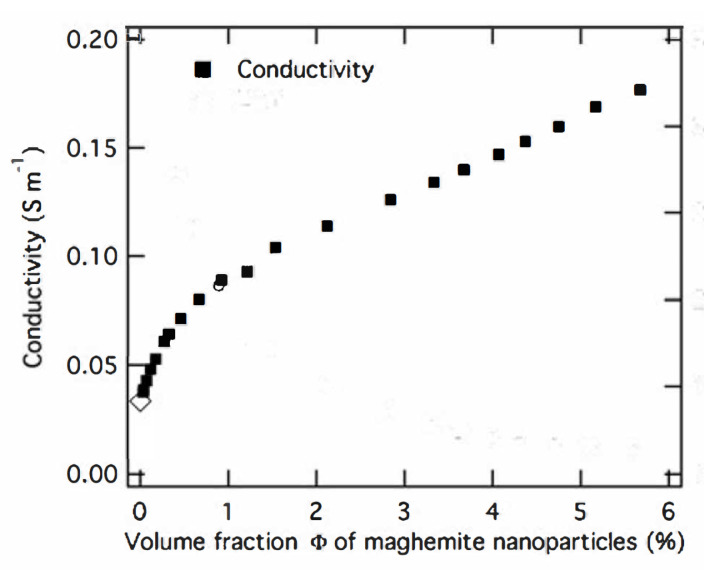
Experimental values of electrical conductivity of water based polyelectrolyte solution as a function of colloidal concentrations. Measurements were performed in pH = 3.1 solutions containing maghemite nanoparticles with an average diameter of 12 nm. More detailed information on the colloidal solution preparation methods and the nature of other ions is found in Ref. [1,2].

Substituting the dipole moment taken in the approximation of Equation (13) in Equation (16) one finds

(17) $$\frac{\Delta \sigma_{\mathrm{CP}}\left(n_{⊙}\right)}{\sigma_{0}}=\frac{\sigma \left(n_{⊙}\right)-\sigma_{0}}{\sigma_{0}}=4\pi n_{⊙}\left(\frac{\gamma -1}{\gamma +2}\right)R^{3},$$

where $\Delta \sigma_{\mathrm{CP}}$ is the excess conductivity due to the presence of colloidal particles. The left-hand-side of this equation can be extracted from the data presented in Figure 2. Indeed, in the interval of the nanoparticles concentrations 0≤ϕ≤0.6% the behavior of conductivity $\sigma (n_{⊙})$ is almost linear and $\sigma \left(n_{⊙}\right)/\sigma_{0}-1=0.7$. In turn, the concentration φ=0.6% corresponds to $n_{⊙}^{\left(1\right)}=5.45\times 10^{15}\;{\mathrm{cm}}^{-3}$.

For further estimations, it will be crucial that Equation (17) is sensitive to the value of γ only when it is not very large. When γ≫1 (we will justify this limit below) the combination (γ−1)/(γ+2)→1 and it ceases to influence the evaluations based on Equation (17). This allows us to find this limit

(18) $$\begin{array}{c} R_{exp}^{\left(1\right)}=2.17\times 10^{-6}\;cm,\\ n_{⊙}^{\left(1\right)}{\left[R_{exp}^{\left(1\right)}\right]}^{3}=0.055\ll 1. \end{array}$$

One can see that these values, together with the nanoparticle concentration $n_{⊙}^{\left(1\right)}$, confirm the validity of the assumed above approximation (1). The plausible reasons for the discovered considerable difference between $R_{exp}^{\left(1\right)}$ and the value of bare radius $R_{0}^{\left(1\right)}=6\times 10^{-7}\;cm$ given in Ref. [1] will be discussed below.

The above found conductivity correction $\Delta \sigma_{\mathrm{CP}}\left(n_{⊙}\right)\propto n_{⊙}R^{3}$ (see Equation (17)) caused by presence of nanoparticles in electrolyte can be confidently distinguished from the standard Onsager–Debye conductivity ($\sigma_{\mathrm{OD}}$) of the diluted 1:1 electrolyte [20,21,22]. Indeed, first of all, the concentration dependencies of these conductivities are different: $\Delta \sigma_{\mathrm{CP}}\left(n_{⊙}\right)\propto n_{⊙}$ while $\sigma_{\mathrm{OD}}\left(n_{⊙}\right)\propto \sqrt{n_{⊙}}$.

Let us focus on the unusual dependence of the excess conductivity (17) of the nanoparticle size: $\Delta \sigma_{\mathrm{CP}}$ growths with increase of *R*. Usually, this dependence is supposed to be opposite (the larger radius of the sphere in Stokes viscous law, the lower its mobility, and hence, the conductivity).

One can analyze the available experimental data on the conductivity of the stabilized diluted colloidal solution [1,2] in the conditions described by Equation (2). In accordance with Equation (17), the excess conductivities for different sizes of nanoparticles in assumption of the same concentration should scale as ${\left[R_{0}^{\left(1\right)}/R_{0}^{\left(2\right)}\right]}^{3}$. Taking the value $R_{0}^{\left(1\right)}=6\;nm$ from [1] and $R_{0}^{\left(2\right)}=3.8\;nm$ from [2] one finds that the ratio

(19) $$\frac{\Delta \sigma_{\mathrm{CP}}^{\left(1\right)}}{\sigma_{0}^{\left(1\right)}}/\frac{\Delta \sigma_{\mathrm{CP}}^{\left(2\right)}}{\sigma_{0}^{\left(2\right)}}={\left(\frac{6}{3.8}\right)}^{3}\approx 4$$

Experimental data for this value give even more striking difference:

(20) $$\frac{\Delta \sigma_{\mathrm{CP}}^{\left(1\right)}}{\sigma_{0}^{\left(1\right)}}/\frac{\Delta \sigma_{\mathrm{CP}}^{\left(2\right)}}{\sigma_{0}^{\left(2\right)}}=\frac{0.7}{0.06}\approx 11.7.$$

### 3.2. Approximation of the Conducting Thick-Walled Spheres

Here, it is necessary to note that the value $R_{exp}^{\left(1\right)}$ obtained in the simple approximation of Equations (13) and (16) and the measured in Ref. [1] bare radius of the colloidal particle $R_{0}$ form a relatively small numerical parameter, ${\left[R_{0}/R_{exp}^{\left(1\right)}\right]}^{3}≃0.02$. It makes sense to improve the experimental data proceeding replacing the value $p_{⊙}$ in Equation (16) by the two parametric expressions (15). Tending γ→∞ in it one finds

(21) $$\frac{\sigma \left(n_{⊙}\right)-\sigma_{0}}{\sigma_{0}}=4\pi n_{⊙}{\left[R_{exp}^{\left(1\right)}\right]}^{3}\left[1-\frac{3}{\gamma }-\frac{9}{2\gamma }\frac{R_{0}^{3}}{\left({\left[R_{exp}^{\left(1\right)}\right]}^{3}-R_{0}^{3}\right)}\right]$$

From this expression, it is clear that the approximation (17) is valid when γ≫1.

The parameter γ requires special discussion. In the DLVO colloidal model, it is assumed that some bare core exists which is able to cause the van der Waals forces between colloidal particles in dilute, non-stabilizing solutions. The conducting properties of this core is not so essential. For example, one can suppose this bare core of the radius $R_{0}$ to be a semiconductor possessing its intrinsic charge carriers which are confined in its volume. If the solvent possesses the stabilizing properties its own mobile charge carriers, counterions have the same properties as the intrinsic charge carriers of the bare core. The requirement of electrochemical potential constancy leads to the charge exchange between the bare core and the solvent. Such exchange results in the formation of the Debye shell (see Equations (4)–(8)), where the concentration of counterions considerably exceeds that in the solvent bulk. We assumed above that the value of corresponding conductivity $\sigma (n_{⊙})$ considerably exceeds $\sigma_{0}$ of the electrolyte conductivity in absence of the nanoparticles. This assumption (γ≫1) breaks when the average value of electrochemical potential in the Debye shell $e\phi_{⊙}$ exceeds the temperature. The authors of Ref. [23] state that in these conditions the Debye shell of the DLVO colloid can crystallize due to Coulomb forces and the latter becomes an insulator with $\sigma \left(n_{⊙}\right)\leq \sigma_{0}$.

## 4. Conclusions

The main result of this work consists of the proposition of an alternative scenario explaining the linear growth of the polyelectrolyte conductivity versus the concentration of colloidal particles observed in Ref. [1,2] in the conditions of the validity of Equation (1). It drastically differs from the existing ideas of the transport in electrolytes resulting in the empirical Kohlrausch’s law (see [22,24])

(22) $$\Delta \sigma ∼\sqrt{n_{⊙}}.$$

The speculations justifying Equation (22) were firstly proposed in early papers such as Ref. [20,21] and the recent efforts to improve this mechanism were undertaken in Ref. [25].

The fact of the observation of the Ohmic transport in strong electrolytes (Ref. [1,2]) denies the applicability of Kohlrausch’s law in the interval of a very low concentration of the colloidal particles. Conversely, the mechanism proposed above, based on the analogy to the percolation mechanism of conductivity occurring in doped semiconductors, allows to get an excellent agreement in the observed linear dependence. Moreover, it also provides very reasonable values of the microscopic parameters of the problem.

One can believe that the validity of Kohlrausch’s law is restored in the domain of higher concentrations and the crossover point between the two regimes (16) and (22) is determined by the condition (2), as is shown in Figure 2. One can find the pro-arguments for this statement in the experimental curve shown in Figure 2 of Ref. [1], where the regimes are changed in the vicinity of the concentration $n_{⊙}^{\left(1\right)}=5.45\times 10^{15}\;cm^{-3}$.

The question that arises is why such linear growth below the percolation threshold was never reported in measurements performed on semiconductors. The answer probably consists of the overwhelming supremacy of the colloidal particle dipole momentum in comparison to that of the dopant in semiconductors.

It would be interesting to compare the values of effective charge *Z* extracted from the experiments on conductivity of [2] and the review article [1]. Unfortunately, this is not easy to do because of the analysis of the data for different *Z* results in very different values of $R_{0}$. It is why one cannot judge the influence of the effective charge *Z* on the bare radius of the colloidal particle $R_{0}$.

The relative insensibility of the polyelectrolyte conductivity on the value of parameter *Z* is not extended on the Seebeck coefficient. The measurements of [2] demonstrate the existence in its kinetics of the two different phases; the initial and steady ones. The authors dealt with two types of colloids; one is almost electroneutral (Z≥1) and the other is supposed to have Z≫1.
